# Molecular Characterization of a New Alkaline-Tolerant Xylanase from *Humicola insolens* Y1

**DOI:** 10.1155/2015/149504

**Published:** 2015-01-05

**Authors:** Pengjun Shi, Yanlong Du, Hong Yang, Huoqing Huang, Xiu Zhang, Yaru Wang, Bin Yao

**Affiliations:** ^1^Key Laboratory for Feed Biotechnology of the Ministry of Agriculture, Feed Research Institute, Chinese Academy of Agricultural Sciences, Beijing 100081, China; ^2^College of Biological Science and Engineering, Bei Fang University of Nationalities, Yinchuan 750021, China

## Abstract

An endo-1,4-*β*-xylanase-encoding gene, *xyn11B*, was cloned from the thermophilic fungus *Humicola insolens* Y1. The gene encodes a multimodular xylanase that consists of a typical hydrophobic signal sequence, a catalytic domain of glycoside hydrolase (GH) family 11, a glycine-rich linker, and a family 1 carbohydrate binding module (CBM1). Deduced Xyn11B shares the highest identity of 74% with a putative xylanase from *Podospora anserina* S mat+. Recombinant Xyn11B was successfully expressed in *Pichia pastoris* and purified to electrophoretic homogeneity. Xyn11B had a high specific activity of 382.0 U mg^−1^ towards beechwood xylan and showed optimal activity at pH 6.0 and 50°C. Distinct from most reported acidic fungal xylanases, Xyn11B was alkaline-tolerant, retaining 30.7% of the maximal activity at pH 9.0. The *K*_*m*_ and *V*_max_ values for beechwood xylan were 2.2 mg mL^−1^ and 462.8 *μ*mol min^−1^ mg^−1^, respectively. The enzyme exhibited a wider substrate specificity and produced a mixture of xylooligosaccharides. All these favorable enzymatic properties make Xyn11B attractive for potential applications in various industries.

## 1. Introduction

Xylan, as a major hemicellulose component of plant cell walls, is the second most abundant polysaccharide after cellulose and accounts for approximately one-third of all renewable organic carbon on earth [1]. Efficient hydrolysis of xylan is an important step towards utilization of abundantly available lignocellulosic materials in nature [2]. Complete breakdown of xylan requires a variety of xylanolytic enzymes, including endo-1,4-*β*-xylanase, *β*-xylosidase, *α*-L-arabinofuranosidase, *α*-D-glucuronidase, acetylxylan esterase, and feruloyl or coumaroyl esterase [3]. Among them, endo-1,4-*β*-xylanase (EC 3.2.1.8) is the crucial enzyme that randomly cleaves the *β*-1,4-glycosidic linkages of the xylan backbone, releasing xylooligomers of different lengths [4]. Xylanases are classified into glycosyl hydrolase (GH) families 5, 7, 8, 10, 11, and 43 based on the similarities of their amino acid sequences (http://www.cazy.org/fam/acc_GH.html) [5].

Microbial xylanases have been applied in many industries, including animal feed, bread-making, paper and pulp, waste treatment, and brewing [6]. Various microorganisms, such as bacteria, yeasts, and fungi, are found to naturally secrete xylanases [7]. Thermotolerant or thermophilic fungi are of industrial and biotechnological interest because they can produce CAZymes that are active at high temperatures and have economic advantages in a wide range of industrial applications [8, 9].

The genus* Humicola* is a well-known nonpathogenic and nontoxic one, which produces a wide variety of hemicellulases and cellulases [10–12]. Thermophilic* Humicola insolens* Y1 has been reported to be an excellent producer of xylanolytic enzymes, including three thermophilic GH 10 xylanases, one GH 11 xylanase (Xyn11A), and two bifunctional GH 43 xylosidase/arabinosidases [13, 14]. In this study, gene cloning and expression of a new GH 11 xylanase gene (*xyn11B*) from* H. insolens* Y1 was reported. This enzyme had distinct properties from Xyn11A and showed high activity at neutral to alkaline pHs.

## 2. Materials and Methods

### 2.1. Strain and Vectors

*Humicola insolens* Y1 CGMCC 4573 (the China General Microbiological Culture Collection Center) was the donor strain [12]. The plasmids pGEM-T Easy (Promega) and pPIC9 (Invitrogen) were used as cloning and expression vectors, respectively. Preparation of all media and protocols for heterologous expression followed the* Pichia* expression manual (Invitrogen).

### 2.2. Chemicals, Reagents, and Kits

The substrates beechwood xylan, 4-nitrophenyl *β*-d-xylopyranoside (*p*NPX), barley *β*-glucan, and carboxymethyl cellulose-sodium (CMC-Na) were purchased from Sigma. Soluble and insoluble wheat arabinoxylan were obtained from Megazyme (Ireland). The DNA purification kit, Genome Walking kit, and LA Taq DNA polymerase were purchased from TaKaRa (Japan). T4 DNA ligase and restriction endonucleases were obtained from New England Biolabs (UK). All other chemicals were of analytical grade and commercially available.

### 2.3. Cloning of the Gene

The full-length GH 11 xylanase gene,* xyn11B*, was identified from the genome sequence of* H. insolens* Y1 (whole genome sequencing in progress). The PCR product was ligated into the pGEM-T Easy vector and transformed into* Escherichia coli* cells for sequencing. The total RNA was extracted from the mycelia after 3 days' growth on wheat bran medium [12] by using the Promega SV Total RNA Isolation System according to the manufacturer's instructions. To obtain the cDNA of the gene* xyn11B*, the first-strand cDNA was synthesized using a ReverTra Ace kit (Toyobo, Japan). The cDNA sequence encoding mature Xyn11B without the signal peptide-coding sequence was amplified using two expression primer sets (*xyn11B*-PF: GAATTCGCCCCCGGTGAGCTGCCTGGCATGC and* xyn11B*-PR: GCGGCCGCTTACAGGCACTGAGAGTACCACTGG, restriction sites underlined). The PCR products with appropriate size were ligated into the pGEM-T Easy vector for sequencing.

### 2.4. Sequence Analysis

Vector NTI 10.0 was used for DNA assembly and sequence analysis. Introns, exons, and transcription initiation sites were predicted using the online software FGENESH (http://linux1.softberry.com/berry.phtml). The signal peptide was predicted by the SignalP 4.0 server [15]. The BLAST server was used for homology searches in GenBank. Multiple alignments of protein sequences were carried out using the ClustalW program [16]. Homology modeling and electrostatic analysis of the catalytic domain were performed with Discovery Studio 2.5 MODELER (Accelrys), using the xylanases from* Chaetomium thermophilum* (PDB: 1XNK) and* Trichoderma longibrachiatum* (PDB: 2JIC) as the templates.

### 2.5. Expression of* xyn11B* in* P. pastoris*

The PCR product was digested with* Eco*RI and* Not*I and cloned into the pPIC9 vector downstream of the *α*-factor signal peptide sequence to construct the recombinant plasmid pPIC9-*xyn11B*. The recombinant plasmid was linearized using* Bgl*II and transformed into* P. pastoris* GS115 competent cells by electroporation. Positive transformants were screened with the xylanase activity assay. The fermentation in shake tubes and 1-L shake flasks was carried out following the method of Liu et al. [17].

### 2.6. Purification of Recombinant Xyn11B

To purify recombinant Xyn11B, the cell-free culture supernatant was collected by centrifugation at 12,000 ×g for 10 min at 4°C, followed by concentration with a Vivaflow 50 ultrafiltration membrane of 5 kDa molecular weight cut-off (Vivascience, Germany). The crude enzyme solution was dialyzed against 20 mM citric acid-Na_2_HPO_4_ (pH 9.0) and loaded onto a HiTrap Q Sepharose XL FPLC column (Amersham Pharmacia Biotech, Sweden) equilibrated with McIlvaine buffer (pH 9.0). Proteins were eluted using a linear gradient of NaCl (0-1.0 M) in the same buffer. Fractions with enzyme activities were collected and subjected to sodium dodecyl sulfate-polyacrylamide gel electrophoresis (SDS-PAGE) as described by Laemmli [18]. The protein concentration was determined by Bradford method [19], using a protein assay kit (Bio-Rad). The purified recombinant Xyn11B was deglycosylated by endo-*β*-*N*-acetylglucosaminidase H (Endo H) at 37°C for 1 h according to the manufacturer's instructions (New England Biolabs).

### 2.7. Xylanase Activity Assay

Xylanase activity was assayed using 3,5-dinitrosalicylic acid (DNS) method [20]. The standard assay mixture contained 900 *μ*L of 1% (w/v) beechwood xylan in 100 mM sodium citrate-phosphate buffer (pH 6.0) and 100 *μ*L of appropriately diluted enzyme. After incubation at 40°C for 10 min, the reaction was terminated by adding 1.5 mL of DNS reagent, boiled for 5 min, and cooled to room temperature. The absorbance was measured at 540 nm. Each assay and its control were done in triplicate. One unit of xylanase activity was defined as the amount of enzyme that released 1 *μ*mol of reducing sugar from the substrate equivalent to xylose per minute under the assay conditions.

### 2.8. Biochemical Characterization

In order to study the biochemical characteristics of purified Xyn11B, buffers of a range of pHs (100 mM citrate buffer, pHs 4–8; 100 mM Tris-HCl, pHs 8-9; and 100 mM glycine-NaOH, pHs 9-10) were used. The optimal pH of Xyn11B for enzyme activity was determined at 40°C for 10 min in buffers mentioned above. Similarly, optimum temperature was determined by assaying the enzyme at different temperatures (20–70°C) and optimum pH for 10 min. For pH-stability assay, the enzyme was preincubated in buffers with a pH range of 4.0–10.0 for 1 h at 37°C, and then the residual activities were examined under standard conditions (pH 6.0, 40°C, and 10 min). To estimate the thermal stability, Xyn11B was incubated in McIlvaine buffer (pH 6.0) at 30°C, 40°C, or 50°C for 5, 10, 15, 20, 30, and 60 min without substrate, and the residual enzyme activity was measured at standard conditions as described above.

To investigate the effects of different metal ions and chemical reagents on Xyn11B activity, the enzymatic activities were measured in McIlvaine buffer (pH 6.0) containing 5 mM of NaCl, KCl, CaCl_2_, LiCl, CoCl_2_, CrCl_3_, NiSO_4_, CuSO_4_, MgSO_4_, FeCl_3_, MnSO_4_, ZnSO_4_, Pb(CH_3_COO)_2_, AgNO_3_, HgCl_2_, EDTA, SDS, or *β*-mercaptoethanol at 40°C for 10 min. Reactions without addition of any chemicals were used as controls.

### 2.9. Substrate Specificity and Kinetic Parameters

Substrate specificity of Xyn11B was estimated in the standard assay system containing 1% (w/v) of beechwood xylan, soluble or insoluble wheat arabinoxylan, barley *β*-glucan, or CMC-Na.

The Michaelis-Menten constant (*K*_*m*_) and the maximum velocity (*V*_max⁡_) of  Xyn11B were calculated by Lineweaver-Burk plot [21]. Each experiment was repeated three times and each experiment included three replicates.

### 2.10. Analysis of Hydrolysis Products

Hydrolysis of beechwood xylan and soluble wheat arabinoxylan by 50 U of Xyn11B was performed in 500 *μ*L of 100 mM citric acid-Na_2_HPO_4_ (pH 6.0) at 37°C for 12 h. After hydrolysis, the enzyme was removed from the reaction using the Nanosep Centrifugal 3 K Device (Pall). The hydrolysis products were analyzed by high-performance anion-exchange chromatography (HPAEC) with a model 2500 system from Dionex. Xylose, xylobiose, xylotriose, and xylotetraose were used as standards.

### 2.11. Nucleotide Sequence Accession Number

The nucleotide sequence of the xylanase gene,* xyn11B*, from* H. insolens* Y1 has been deposited in the GenBank database under accession number KM275236.

## 3. Results

### 3.1. Gene Cloning and Sequence Analysis

One xylanase coding gene,* xyn11B*, 995 bp in length, was identified in the genome sequence of* H. insolens* Y1. The cDNA of* xyn11B* contains 876 bp and encodes 291 amino acids. The deduced Xyn11B contains a putative N-terminal signal peptide at residues 1–19, a typical catalytic domain of GH 11 (residues 20–222), a glycine-rich linker at residues 223–255 (18/32 glycine residues), and a family 1 carbohydrate binding module (CBM1) at residues 256–291. The molecular weight and* p*I value of mature Xyn11B were estimated to be 29.1 kDa and 8.7, respectively.

**Figure 1:**
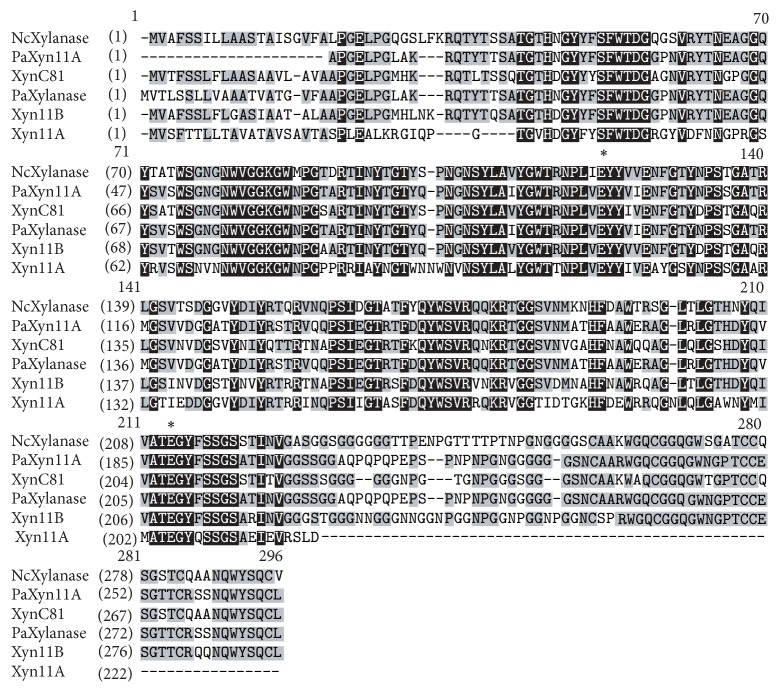
Multiple sequence alignment of Xyn11B with other GH 11 endo-*β*-14-xylanases. PaXylanase from* Podospora anserina* S mat+ (XP_001903201.1), XynC81 from* Achaetomium* sp. Xz-8 (AHE13929.1), PaXyn11A from* P. anserina* (ADO14136.2), NcXylanase from* Neurospora crassa* (CAD71059.1), and Xyn11A from* Humicola insolens* Y1 (KC962401). Identical and similar amino acids are indicated by solid black and gray shades, respectively. The two catalytic glutamate residues are indicated by asterisks.

Multiple sequence alignment of Xyn11B with available protein sequences from GenBank (Figure 1) indicated that Xyn11B shares the highest identity of 74% to a putative GH 11 xylanase from* Podospora anserina* S mat+ (XP_001903201.1), followed by the functionally characterized GH 11 xylanase XynC81 from* Achaetomium* sp. Xz-8 (AHE13929.1, 76%). The putative tertiary structure of Xyn11B catalytic domain displays the classical *β*-jelly-roll architecture, and two catalytic residues (Glu118 and Glu209) and consensus sequence PSIXG (X corresponds to a nonconserved residue) were identified. Solvent-exposed amino acid analysis revealed a high frequency of positively charged residues on the surface. In addition, Xyn11B contains two potential* N*-glycosylation sites (Asn93 and Asn255).

### 3.2. Expression, Purification, and Deglycosylation of Recombinant Xyn11B

**Figure 2:**
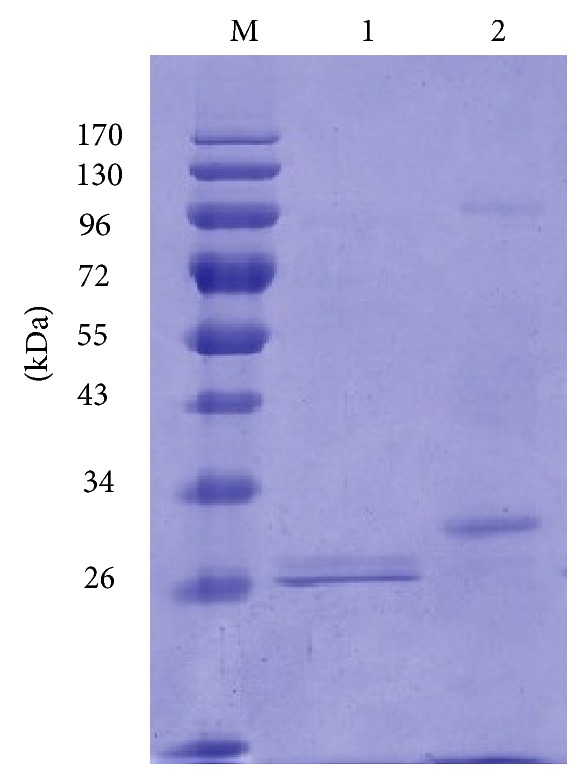
SDS-PAGE analysis of purified recombinant Xyn11B. Lanes: (M) the standard protein molecular weight markers; (1) the purified recombinant Xyn11B after deglycosylation with Endo H; and (2) the purified recombinant Xyn11B.

The gene fragment coding for mature Xyn11B was cloned into vector pPIC9 and successfully expressed in* P. pastoris*. After methanol induction for 72 h, the transformant with highest xylanase activity showed the activity of 101.5 U mL^−1^ in the culture supernatant. Recombinant Xyn11B was purified to electrophoretic homogeneity by a single-step anion-exchange chromatography (Figure 2). The specific activity of the purified recombinant Xyn11B was 382.0 U mg^−1^ towards beechwood xylan. The apparent molecular mass of purified recombinant Xyn11B on SDS-PAGE gel was found to be about 32.0 kDa, which was higher than its theoretical molecular mass (29.1 kDa). After deglycosylation with Endo H, the purified recombinant Xyn11B migrated as a single band of ~29.0 kDa, which was in agreement with the calculated molecular weight. Purified enzyme was identified by size-exclusion chromatography as monomer of 32.0 kDa without carbohydrate residue.

### 3.3. Enzyme Characterization

**Figure 3:**
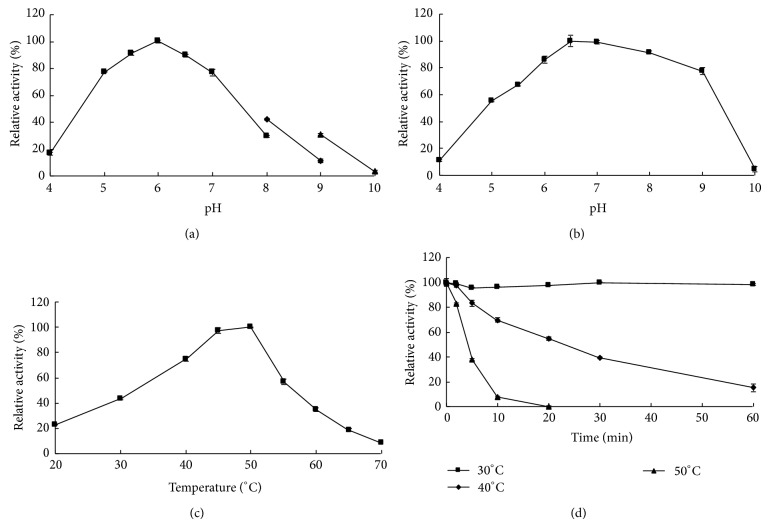
Characterization of purified recombinant Xyn11B. (a) Effect of pH on enzyme activity. (b) pH stability of Xyn11B. (c) Effect of temperature on enzyme activity. (d) Thermostability assay at 30°C, 40°C, or 50°C. Each value in the panel represents the means ± SD (*n* = 3).

Purified recombinant Xyn11B had a pH optimum of 6.0 at 40°C (Figure 3(a)). It showed good pH adaptability, retaining more than 40% of the maximal activity at pH 5.0–8.0 and more than 30% activity at pH 9.0. For the pH stability of Xyn11B, it was stable at pH 5.0–9.0, retaining almost 80% of the maximal activity after incubation at 37°C for 1 h (Figure 3(b)). The optimal temperature for Xyn11B activity was 50°C (Figure 3(c)). In the thermostability assay, Xyn11B was stable at 40°C and below and lost almost all of its activity at 50°C for 20 min (Figure 3(d)).

The effect of different metal ions or chemical reagents on the activity of purified Xyn11B was determined (Table 1). Addition of most metal ions and EDTA at the concentration of 5 mM had little effect on the activity of Xyn11B. SDS and Hg^2+^ were strong inhibitors for Xyn11B activity. On the other hand, the activity of Xyn11B was significantly enhanced in the presence of *β*-mercaptoethanol.

### 3.4. Substrate Specificity and Kinetic Parameters

Xyn11B showed maximum activity on soluble wheat arabinoxylan (defined as 100%), moderate activity on birchwood xylan (80.5%) and beechwood xylan (76.2%), and weak activity on insoluble wheat arabinoxylan (7.0%). No activity was detected in the presence of barley *β*-glucan or CMC-Na.

Using beechwood xylan as the substrate, the* K*_*m*_ and *V*_max⁡_ values were 2.2 mg mL^−1^ and 462.8 *μ*mol min^−1 ^mg^−1^, respectively.

### 3.5. Analysis of Hydrolysis Products

The products of beechwood xylan and soluble wheat arabinoxylan by Xyn11B hydrolysis were analyzed by HPAEC. The main products of beechwood xylan were 2.3% xylose, 25.1% xylobiose, 52.2% xylotriose, and 20.4 xylotetraose. The composition of the hydrolysis products from soluble wheat arabinoxylan was 50.5% xylose and 49.5% xylobiose.

## 4. Discussion

In this study, a new GH 11 xylanase from* H. insolens* Y1 was identified, cloned, and successfully expressed in* P. pastoris*. Xyn11B is a multimodular enzyme that contains four regions: an N-terminal leader sequence, a GH 11 catalytic domain, a glycine-rich linker, and a C-terminal CBM1. To our knowledge, about 25% of functionally characterized GH 11 xylanases carry one CBM; among them are the most characterized fungal GH 11 xylanases bearing a CBM1 module, which assists the catalytic domain to bind to insoluble and crystalline polysaccharides [22, 23]. When using beechwood xylan as the substrate, CBM-truncated version of Xyn11B had similar enzymatic properties as the wild-type one but had no activity against insoluble wheat arabinoxylan (data not shown). The results indicated the importance of CBM1 in enzyme activity on insoluble substrate.

Most fungal GH 11 xylanases are acidic and have pH optima below 5.5 [24], and some of them are even acidophilic (Table 2). For example, XynC from* Aspergillus kawachii* and XynA from* Penicillium* sp. 40 have optimal pHs at 2.0 [25, 26], and XYL11B from* Bispora* sp. MEY-1 has an optimal pH at 2.6 [27]. There are exceptions that have neutral pHs [24]. Xyn11B and Xyn11A [14] from* H. insolens* Y1 have optimal pHs at 6.0 and 7.0, respectively, and are both alkali-tolerant, exhibiting 50.6% and 30.7% of the maximum activities at pH 9.0, respectively. From the same strain, another three GH 10 xylanases were also found to be alkali-tolerant [13]. The mechanism of* H. insolens* Y1 xylanases with higher activities at alkaline pHs requires further study. In addition, Xyn11B had maximal activities at 50°C, lower than Xyn11A (60°C). Its weak thermostability (only stable at 30°C and below) is similar to other GH 11 xylanases from mesophilic fungi, such as PfXynC from* P. funiculosum* [28], XYN11F63 from* Penicillium* sp. F63 [17], and XylA and XylB from* Fusarium graminearum* [29].

Like most GH 11 xylanases, Xyn11B activity was completely inhibited by Hg^2+^, a metal ion that interacts with Trp and oxidizes the indole ring [30], and was significantly enhanced by *β*-mercaptoethanol, which counteracts the oxidation effects of the S–S linkage between Cys residues [31]. Interestingly, addition of SDS leads to the complete activity loss of most fungal GH 11 xylanases, but not Xyl11B. In the presence of 5 mM of SDS, XynC retained 28.2% activity. The SDS tolerance and neutral to alkaline preference are important for application in the detergent and textile solutions. On the other hand, Xyn11B can hydrolyze commercial xylans and has no activity towards glucan or CMC-Na. These alkaline-active, cellulose activity-free characteristics make Xyn11B preferred for biobleaching of paper pulp [30, 31].

Xyn11B had higher activity on soluble wheat arabinoxylan than beechwood xylan and birchwood xylan. Based on the HAPEC analysis, the hydrolysis products of wheat arabinoxylan compared to beechwood xylan were different. The hydrolysis products from soluble wheat arabinoxylan were equal amounts of xylose and xylobiose. However, the beechwood xylan products were a mixture of xylose, xylobiose, xylotriose, and xylotetraose. In the viewpoint of xylan composition, beechwood xylan and birchwood xylan have similar compositions (89% xylose and 1% arabinose), but soluble wheat arabinoxylan contains 38% arabinose and is more complex in side chain structure [8, 14, 15]. Xyn11B may cleave the glycosidic linkages of main chains closer to substituents in soluble wheat arabinoxylan than in beechwood, thus yielding smaller hydrolysis products. The real reason needs more detailed research.

## 5. Conclusions

In summary, Xyn11A and Xyn11B produced by* H. insolens* Y1 are both alkali-tolerant but have different temperature optima (60°C and 50°C, resp.) and thermostability. Xyn11B has complex hydrolysis products from various xylan substrates. These superior properties make Xyn11B a good candidate for the xylooligosaccharide production.

## Figures and Tables

**Table 1:** Effect of 5 mM metal ions and chemical reagents on the xylanase activity of Xyn11B.

Chemicals	Relative activity (%)^a^	Chemicals	Relative activity (%)
None	100.0 ± 1.2	Ca^2+^	95.7 ± 1.2
Mn^2+^	110.4 ± 2.8	Cr^3+^	93.0 ± 0.6
Na^+^	101.6 ± 1.3	Pb^2+^	91.6 ± 1.8
Li^+^	100.6 ± 1.4	Cu^2+^	87.0 ± 2.3
Co^2+^	100.6 ± 0.9	Ag^+^	76.5 ± 2.1
Zn^2+^	99.6 ± 2.2	Hg^2+^	0
K^+^	99.2 ± 0.4	*β*-Mercaptoethanol	160.6 ± 4.7
Fe^3+^	98.7 ± 1.6	EDTA	96.1 ± 1.1
Mg^2+^	96.6 ± 0.8	SDS	28.2 ± 3.2

^a^Values represent the mean ± SD (*n* = 3) relative to untreated samples.

**Table 2:** Property comparison of Xyn11B with family 11 xylanases representatives.

Microorganisms	Xylanase name	MW (kDa)	pH_opt_	T_opt_ (°C)	Residual activity or thermostability	References
*Humicola insolens* Y1	Xyn11B	29.1	6.0	40	Stable at 40°C	This study
*Humicola insolens* Y1	Xyn11A	22.8	7.0	60	Stable at 50°C	[14]
*Penicillium* sp. CGMCC 1669	XYN11F63	21.5	4.5	40	91.7% after 1 h at 40°C	[17]
*Aspergillus kawachii *	XynC	20.0	2.0	50	ND^a^	[25]
*Penicillium *sp. 40	XynA	20.7	2.0	50	Stable at 30°C	[26]
*Bispora* sp. MEY-1	XYL11B	23.1	2.6	65	70% after 60 min at 70°C	[27]
*Penicillium griseofulvum *	PgXynA	20.8	5.5	50–60	Stable at 30°C	[28]
*Penicillium funiculosum *	PfXynC	21.1	5.5	50–60	Stable at 30°C	[28]
*Fusarium graminearum *	XylA	24.0	8.0	35	20% activity at 45°C	[29]
*Fusarium graminearum *	XylB	22.7	7.0	35	34% activity at 45°C	[29]

^a^ND: not determined.
